# Biosecurity and Yield Improvement Technologies Are Strategic Complements in the Fight against Food Insecurity

**DOI:** 10.1371/journal.pone.0026084

**Published:** 2011-10-12

**Authors:** David C. Cook, Rob W. Fraser, Dean R. Paini, Andrew C. Warden, W. Mark Lonsdale, Paul J. De Barro

**Affiliations:** 1 Department of Agriculture and Food Western Australia, Bunbury, Western Australia, Australia; 2 Cooperative Research Centre for National Plant Biosecurity, Bruce, Australian Capital Territory, Australia; 3 Department of Economics, The University of Kent, Canterbury, Kent, United Kingdom; 4 CSIRO Ecosystem Sciences, Canberra, Australian Capital Territory, Australia; Cairo University, Egypt

## Abstract

The delivery of food security via continued crop yield improvement alone is not an effective food security strategy, and must be supported by pre- and post-border biosecurity policies to guard against perverse outcomes. In the wake of the green revolution, yield gains have been in steady decline, while post-harvest crop losses have increased as a result of insufficiently resourced and uncoordinated efforts to control spoilage throughout global transport and storage networks. This paper focuses on the role that biosecurity is set to play in future food security by preventing both pre- and post-harvest losses, thereby protecting crop yield. We model biosecurity as a food security technology that may complement conventional yield improvement policies if the gains in global farm profits are sufficient to offset the costs of implementation and maintenance. Using phytosanitary measures that slow global spread of the Ug99 strain of wheat stem rust as an example of pre-border biosecurity risk mitigation and combining it with post-border surveillance and invasive alien species control efforts, we estimate global farm profitability may be improved by over US$4.5 billion per annum.

## Introduction

While there is general agreement on the increased global demand for food to be expected in the coming decades, there is uncertainty surrounding global agriculture's capacity to service this demand through an expansion in the food supply. On a global scale, yield growth amongst major cereal crops has generally declined since the green revolution of the 1960s and 1970s, while the total area under cultivation has remained constant [1]–[3]. About one per cent (50 000 km^2^) of farm land is lost annually to the effects of degradation, desertification, urban sprawl, mining, recreation, toxic pollution and rising sea levels [4]. In contrast, the human population is expected to rise to 9.2 billion by 2050 from about 6.7 billion in 2008 [5].

Despite the need to capitalise on declining yield gains to feed these future populations, global crop losses caused by introduced pests and diseases continue to increase around the world [6]. We refer to these species as Invasive Alien Species (IAS), using the term to describe introduced pathogen, pest or weed species that have a net negative effect on social welfare as determined by environmental, economic and social capital [7]. Without management controls, it is estimated that IAS have the potential to inflict pre-harvest yield losses ranging from 44–54 per cent in wheat, 64–80 per cent in rice, 58–75 per cent in maize, 73–80 per cent in potatoes and 49–69 per cent in soybeans [8]. Even with controls, losses average 28 per cent in wheat, 37 per cent in rice, 31 per cent in maize, 40 per cent in potatoes and 26 per cent in soybeans [8].

To give some examples, the fungal pathogen Black Sigatoka (*Mycosphaerella fijiensis*) can reduce banana yields by 50 per cent [9]. The fungal pathogen Rice Blast (*Magnaporthe oryzae*) has been estimated to causes production losses sufficient to feed 60 million people per year [9]. Rodents consume approximately 6 per cent of the annual Indonesian rice harvest, which is sufficient to feed Indonesia's 240 million people for a year [10], [11]. Witchweed *Striga hermonthica* has invaded 20–40 million hectares of arable land in sub-Saharan Africa and reduced crop yields by more than 20 per cent [8], [10]. Weeds in general have the potential to reduce global wheat yields by 18–29 per cent [8].

Cereals are the most prevalent group of crops grown across the world, and as such IAS that affect or destroy cereal yields pose a particular threat to *food security*. While many definitions of this term have been put forward (see Maxwell [12] for 32 separate definitions), common to most are the underlying themes of food availability; consumer access to affordable, nutritional and safe food; resilience of the food system to disruptions; and public confidence in that system. Collectively, cereals cultivation takes place over 61 per cent of the total cultivated land, over a third of which is wheat, making it an important factor in the food security of populations worldwide [13]. Wheat is grown across the Great Plains of the United States, the Canadian Prairie Provinces, the Indus and the upper Ganges Valleys, along the Kazakhstan and Russian border and southern Australia [13]. Although many exotic species have the capacity to damage crops in these regions, the consequences of a new wheat stem rust (*Puccinia graminis*) epidemic may be catastrophic [9], [14]. With the discovery in 1999 of a new race of the stem rust fungal pathogen in Uganda (known as Ug99) capable of overcoming existing stem rust resistance, this is a very real prospect [15].

In this paper we examine the role of both pre-border biosecurity measures that reduce the likelihood of IAS like Ug99 crossing national borders, and post-border policies that lead to the early detection of border breaches and subsequent management once they have established and spread widely. While acknowledging the evolution of thought from the supply-oriented first generation view of food security to a more complex third generation view [16]–[21], we apply a simplistic first generation view and focus on the benefits of investments aimed at reducing the spread and impact of IAS of wheat (hereon referred to as ‘biosecurity’) and crop breeding technologies that increase global wheat supply. We treat this investment as a new food security technology and investigate its potential for global adoption if the incentives of the top wheat-producing countries are aligned towards the maximisation of joint production over time.

## Methods

We treat biosecurity as an investment alternative to conventional yield-increasing technologies for a fictitious central planner with a first generation view of food security. That is, provision of food security (and prevention against food insecurity) is purely a matter of supply management. The central planner is able to dictate investment in food security achieving activities across a number of different countries. Predicted investment paths across these countries are defined as a function of expected yield and cost changes (and hence profitability) from investing in biosecurity relative to yield-increasing crop varieties. We make the assumption that the central planner will choose to invest in biosecurity measures against the threat posed by Ug99 in country *i* in time step (i.e. year) *t* if it is expected to reduce grower losses by a greater amount than additional costs. The dichotomous adoption variable, 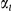,which takes on the value of 1 if the central planner invests in biosecurity across *n* countries in year *t* and zero otherwise, is defined as:
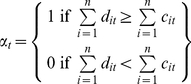
(1)where 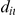 is the total difference in predicted cost increments induced by Ug99 between biosecurity measures and yield improvement technologies in country *i* in time *t*, and 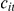 is the total cost of implementing pre- and post-border biosecurity measures in country *i* in time *t*. We focus on the estimation of 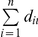 to determine how large 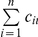 would need to be before 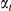 assumes a value of 0. Therefore, the nature and effectiveness of biosecurity measures are paramount.

The current international pre-border biosecurity strategy for addressing the threat of Ug99 includes the use of phytosanitary measures on traded wheat and wheat products, which lower the probability of the rust spreading via trade routes. Post-border biosecurity measures include monitoring through intensive disease surveillance, and rapid, sensitive and robust detection leading to early warning and rapid incursion responses and risk mitigation strategies [22]. These are complemented by storage hygiene, sampling and fumigation measures that are effective against a wide range of storage IAS.

If, as a result of these post-border measures, a Ug99 infection is detected early enough, there may be a strong likelihood of a fast eradication through crop removal and destruction. Hence, the value of 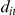 is influenced by eradication costs and probability of success, as well as the capacity of countries to capitalise on this eradication and prevent post-harvest losses.

If an outbreak is not detected early enough, a longer term management strategy is required to minimise the rust's impacts using crop technologies and chemical treatments. For this longer term management to be successful, the development and release of resistant cultivars is essential to reduce vulnerability to the disease and its further spread throughout wheat growing regions. While mildly resistant varieties are available in the short term, the long-term strategy involves redeveloping the Sr2-complex which combines the slow rusting gene Sr2 with additional rust resistant genes to achieve prolonged resistance [23], [24].

Algebraically, we expressed 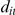 as:
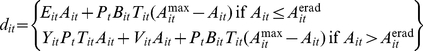
(2)where: 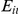 is the cost of eradication per hectare in country *i* in year *t*; 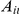 is the area infected with Ug99 in country *i* in year *t* weighted by the probability of infection and density of infection; 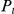 is the world price for wheat in year *t*; 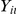 is the change in yield resulting from replanting to lower-yielding rust resistant wheat varieties in country *i* in year *t*; 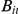
is the average wheat yield in country *i* in year *t*; 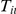 is the proportion of crop lost post-harvest during storage and transport to stored grain IAS in country *i* in year *t*; 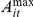 is the total area of wheat grown in country *i* in year *t*; 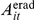 is the maximum technically feasible area of eradication in country *i* in year *t*; and 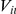 is the increase in variable cost of production per hectare induced by Ug99 on-farm management methods in country *i* in year *t*.


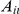
 contains a great deal of biological information. It is inclusive of entry and establishment probabilities (denoted 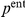 and 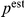, respectively), and therefore represents the area predicted to be in need of additional management effort (i.e. beyond normal farm management activities) due to Ug99 infection in country *i* in year *t*. A Markov chain process, described in Hinchy and Fisher [25], is used to change 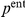 and 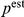 over time according to a vector of transitional probabilities. These transitional probabilities describe the likelihood of moving from one pest/disease state to another. 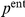 and 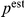 are combined to form a probability of invasion, 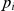:
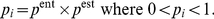
(3)


To describe the movement of Ug99 post-establishment in multiple countries we use a stratified diffusion model combining both short and long distance dispersal processes [26]. It is derived from the reaction diffusion models originally developed by Fisher [27] which have been shown to provide a reasonable approximation of the spread of a diverse range of organisms [28]–[31]. These models assert that an invasion diffusing from a point source will eventually reach a constant asymptotic radial spread rate of 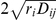 in all directions, where 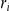 describes a growth factor for Ug99 per year in country *i* (assumed constant over all infected sites) and 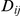 is a diffusion coefficient for an infected site *j* in country *i* (assumed constant over time) [32]–[34]. Hence, we assume that the original infection (i.e. the first of a probable series of sites, *j*) takes place in a homogenous environment in country *i* and expands by a diffusive process such that area infected at time *t*, 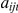, can be predicted by:
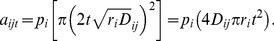
(4)For practical purposes, an estimate of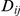 can be derived from the mean dispersal distance (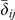) of the pathogen at an infection site, where 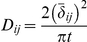 [35]. 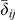 is the site-specific average distance (in metres) over which dispersal events leading to infection occur. By assuming 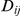 is constant across all sites *j* we ignore demographic stochasticity and consequent non-uniform invasion.

The density of Ug99 infection within 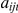 influences the control measures required to counter the effects of infection, and thus partially determines the value of 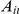. We assume that in each site *j* in country *i* affected, the infection density, 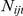, grows over time period *t* following a logistic growth curve until the carrying capacity of the environment, 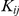, is reached:
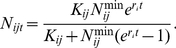
(5)Here, 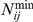 is the size of the original influx in region *j* of country *i* and 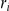 is the intrinsic rate of density increase in country *i* (assumed to be the same as the intrinsic rate of population increase).

In addition to 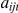 and 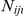, the size of 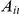 depends on the number of nascent foci (see Moody and Mack [36] – these are *satellite* infection sites) in year *t*, 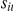, which can take on a maximum value of 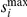 in any year. These sites result from events external to the outbreak itself, such as weather phenomena, animal or human behaviour, which periodically jump the expanding infection beyond the infection front. We use a logistic equation to generate changes in 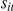 as an outbreak continues:
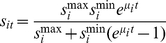
(6)where 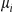 is the intrinsic rate of new foci generation in country *i* (assumed constant over all *t*), and 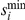 is the minimum number of satellite sites generated in country *i*.

Given equations (4)–(6), we can express 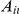 as:
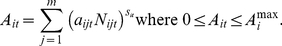
(7)The total benefit to the central planner in terms of the alleviation of global food insecurity through biosecurity in year *t*, 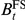, can be expressed as:
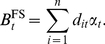
(8)Recall that the central planner maintains a first generation view of food security and is motivated by policies that affect global wheat supply.

In the following section we estimate 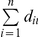 using multiple Ug99 spread scenarios for the top 15 wheat producing countries of the world (i.e. 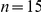) over a 30 year period (see Table 1). Where there is uncertainty surrounding parameter values, they are specified within the model as distributions and a Latin hypercube sampling algorithm used to sample from each distribution. In each of 10 000 model iterations one value is sampled from the cumulative distribution function so that sampled parameter values are weighted according to their probability of occurrence. The model calculations are then performed using this set of parameters.

**Table 1:** International wheat production statistics, labour costs and Ug99 establishment indexes by country.

Producer	Area planted to wheat (ha) a	Mass of grain produced (MT) a	Average yield (T/ha) a	Value produced (US$'000,000) a	Labour rate (US$/hr) b	Ug99 establishment index c
China	24,210,075	114,950,296	4.75	17,395	5.00	0.134360
India	28,400,000	78,570,200	2.77	11,614	4.00	0.134360
United States of America	20,181,081	68,016,100	3.37	8,775	26.35	0.024677
Russian Federation	26,632,900	63,765,140	2.39	5,738	4.91	0.019464
Canada	9,539,000	28,611,100	3.00	3,529	29.20	0.024677
France	5,146,600	39,001,700	7.58	4,141	30.93	0.024677
Pakistan	9,046,000	20,958,800	2.32	3,040	4.34	0.181200
Australia	13,507,000	21,420,177	1.59	2,308	35.00	0.134360
Ukraine	6,752,900	25,885,400	3.83	1,795	4.01	0.002024
Turkey	8,026,898	17,782,000	2.22	2,660	12.35	0.113270
Germany	3,226,036	25,988,565	8.06	2,067	35.00	0.024677
United Kingdom	1,814,000	17,227,000	9.50	1,273	39.49	0.024677
Kazakhstan	14,329,400	12,538,200	0.87	1,358	2.52	0.000267
Argentina	4,334,780	8,508,156	1.96	2,034	16.03	0.134360
Egypt	1,321,751	7,977,051	6.04	992	5.00	0.120550

^*a*^FAO [46];

^*b*^Based on hourly wages (US$) for rural workers from U.S. Department of State [47];

^*c*^Derived from Paini et al. [37].

Table 1 provides wheat production information for each country used in the analysis and approximate labour costs (used below in forming estimates of eradication and control costs). It also contains country-specific Ug99 establishment likelihood indexes derived from Self Organising Map (SOM) analysis, which is a type of artificial neural network. This technique uses worldwide species associations to determine which species have the highest likelihood of establishing in a particular region. Paini et al. [37] performed a SOM analysis on the worldwide distribution of 131 plant pathogens, including the Ug99 race of *P. graminis*. The index values produced for each country are used as a proxy for establishment probabilities.

A list of all the model parameter distributions appears in Table 2. Note that *i*, *j* and *t* subscripts are omitted since, with the exception of *p* ^ent^ and increased chemical cost, parameter specification does not change over spatial or temporal ranges. Table notes provide details where a spatial variation is assumed.

**Table 2:** Parameter estimates.

Parameters	With Biosecurity Measures	Without Biosecurity Measures
Probability of entry, *p* ^ent^. a	Uniform(1.0×10^−6^, 1.0×10^−3^)	Uniform(0.3,0.7)
Probability of establishment, *p* ^est^. b	2.6×10^−4^ to 1.3×10^−1^	2.6×10^−4^ to 1.3×10^−1^
Detection probability.	Binomial(1.0, 0.5)	Binomial(1.0,0.3)
Probability of successful eradication in a single time step given an infected area, *A*, and a maximum area considered for eradication, *A* ^erad^ (see below).	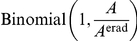	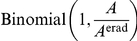
Population diffusion coefficient, *D* (m^2^/yr). a ^,^ c	Pert(0,2.5×10^3^, 5.0×10^3^)	Pert(0,2.5×10^3^,5.0×10^3^)
Minimum area infected immediately upon entry, *A* ^min^ (m^2^).	1.0×10^3^	1.0×10^3^
Maximum area infected, *A* ^max^ (m^2^). d	1.8×10^12^	1.8×10^12^
Intrinsic rate of infection and density increase, *r*(yr^−1^). a	Pert(1.0,1.25,1.5)	Pert(1.0,1.25,1.5)
Minimum infection density, *N* ^min^ (#/m^2^).	1.0×10^−4^	1.0×10^−4^
Maximum infection density, *K* (#/m^2^). a	Pert(100,550,1000)	Pert(100,550,1000)
Minimum number of satellite sites generated in a single time step, *S* ^min^ (#).	1	1
Maximum number of satellite sites generated in a single time step, *S* ^max^ (#). a	Pert(70,85,100)	Pert(70,85,100)
Intrinsic rate of new foci generation per unit area of infection, *µ* (#/m^2^). a *^,^* c	Pert(1.0×10^−6^,3.0×10^−6^,5.0×10^−6^)	Pert(1.0×10^−6^,3.0×10^−6^,5.0×10^−6^)
Discount rate (%).	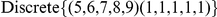	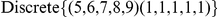
Supply elasticity. e	Pert(0.2,0.3,0.4)	Pert(0.2,0.3,0.4)
Demand elasticity. e	Pert(-0.2,-0.3,-0.4)	Pert(-0.2,-0.3,-0.4)
World wheat price in the first time step (US$/T). d *^,^* f	Uniform(155,275)	Uniform(155,275)
Average yield, *B* (T/ha). d	0.87 to 9.50	0.87 to 9.50
Maximum area considered for eradication (ha).	10 000	10 000
Increased chemical cost (US$/ha). a	40	40
Increased application costs (US$/ha). g	2.50 to 39.50	2.50 to 39.50
Cost of eradication, *E* (US$/ha). h	Pert(5.0×10^3^,1.0×10^4^,1.5×10^4^)	Pert(5.0×10^3^,1.0×10^4^,1.5×10^4^)
Yield reduction from adoption of resistant varieties, *Y* (%).	Pert(5,10,15)	Pert(5,10,15)
Post-harvest loss (%). i	Pert(21,30,39)	Pert(30,35,40)

^*a*^Specified with reference to Cook [48] and Waage et al. [49] using distributions defined in Biosecurity Australia [50];

^*b*^See country-specific Ug99 establishment indexes in Table 1 derived from Paini et al. [37] and interpreted here as establishment probabilities;

^*c*^Derived from Sapoukhina et al. [51];

^*d*^FAO [46]. Note 1ha  =  10 000 m^2^ ;

^*e*^Specified with reference to FAPRI [52];

^*f*^International Monetary Fund [53];

^*g*^Based on time taken for crop removal (see *^h^*, below) and hourly wages (US$) for rural workers from U.S. Department of State [47] provided in Table 1;

^*h*^Assumes zero compensation following crop destruction, and transport, disposal and chemical costs amounting to US$10,800 per hectare. This is inclusive of labour (see Table 1), machinery ($100/hr at approximately 20 minutes per hectare depending on yield, soil, terrain, etc.), truck hire ($75/hr), incendiaries ($6/ha for green waste) and creation of a circular chemical buffer zone approximately 10 hectares in diameter around previously infected sites. Chemical used is assumed to be *Folicur 430* (or equivalent, e.g. *Impact 250*, *Tilt 250* or *Triad 125*) applied at a rate of 145–290 mL/ha, costing $20–40/ha and taking 6 minutes per hectare to apply);

^*i*^Estimate without biosecurity measures derived from Oerke and Dehne [6] and Oerke [8], while the with biosecurity measures estimate implies an arbitrary reduction in post-harvest losses of Pert(1%, 5%, 9%).

## Results

The present value of average benefits accruing from pre-border, border and post-border biosecurity activities specifically targeted at Ug99 is estimated by the model to average US$4.5 billion per year across the 15 wheat producing countries used in the assessment (i.e. 
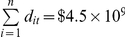
). Recall from equations (1) and (8), this represents the threshold level of 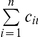 beyond which the central planner will choose not to invest in pre- and post-border biosecurity as an alternative to a traditional yield-oriented food security strategy (i.e. 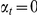). The standard deviation of the distribution of average annual biosecurity benefits is US$2.8 billion and skewness 1.4 (i.e. the distribution is skewed right such that the right tail is long compared to the left tail).

Given current average wheat yields, our estimated value of 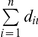 is equivalent to an annual increase in the global wheat harvest volume of 46.8 million tonnes per year. To achieve an equivalent supply increase through crop breeding average yields would need to increase by approximately 7 per cent per year over the same period, which exceeds all gains achieved from the past forty years of crop breeding and engineering. Production data suggests that wheat yield growth in the developed world has averaged just over 1 per cent per annum since 1965 [38]–[40], while less developed production regions have experienced 2–3 per cent annual yield improvements since 1979 [38], [41], [42]. This amounts to an additional 4–25 million tonnes of wheat produced annually at an estimated value of US$2–6 billion [38]–[40].

**Figure 1:**
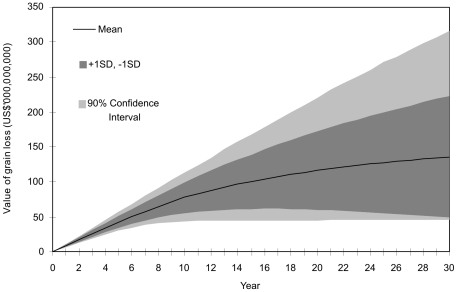
Cumulative benefit of biosecurity measures to mitigate the spread of Ug99 throughout prominent wheat production areas of the world over time.

Over a 30-year period, the mean benefit of pre- and post-border biosecurity predicted by the model is US$136.4 billion, but the uncertainty in projecting this far into the future is reflected in a standard deviation of US$86.3 billion. The variability of results predicted over time is illustrated in Figure 1 where the mean cumulative benefit predicted by the model is plotted ± 1 standard deviation and 5 per cent and 95 per cent confidence bounds.

If we separate our estimate of 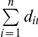 into its pre- and post-border biosecurity components, we find that the largest returns to investment occur through post-border biosecurity. This is shown in Table 3, which provides the mean and standard deviation of predicted annual benefits with pre- and post-border measures, and combined. While pre-border biosecurity benefits are equivalent to a 1 per cent increase in average wheat yields (or an additional 7.6 million tonnes harvested per annum), far greater gains are possible through post-border biosecurity measures. While there is a large amount of uncertainty surrounding each of these estimates, indicated by large standard deviations, particularly with respect to the combined total benefit, the effects of post-border measures dominate those of pre-border biosecurity measures.

**Table 3:** Annual pre-border, post-border and combined biosecurity benefits.

	Pre-Border (US$ billion)	Post-Border (US$ billion)	Combined (US$ billion)
Mean	0.7	4.2	4.5
St. Dev.	0.7	2.2	2.9

**Figure 2:**
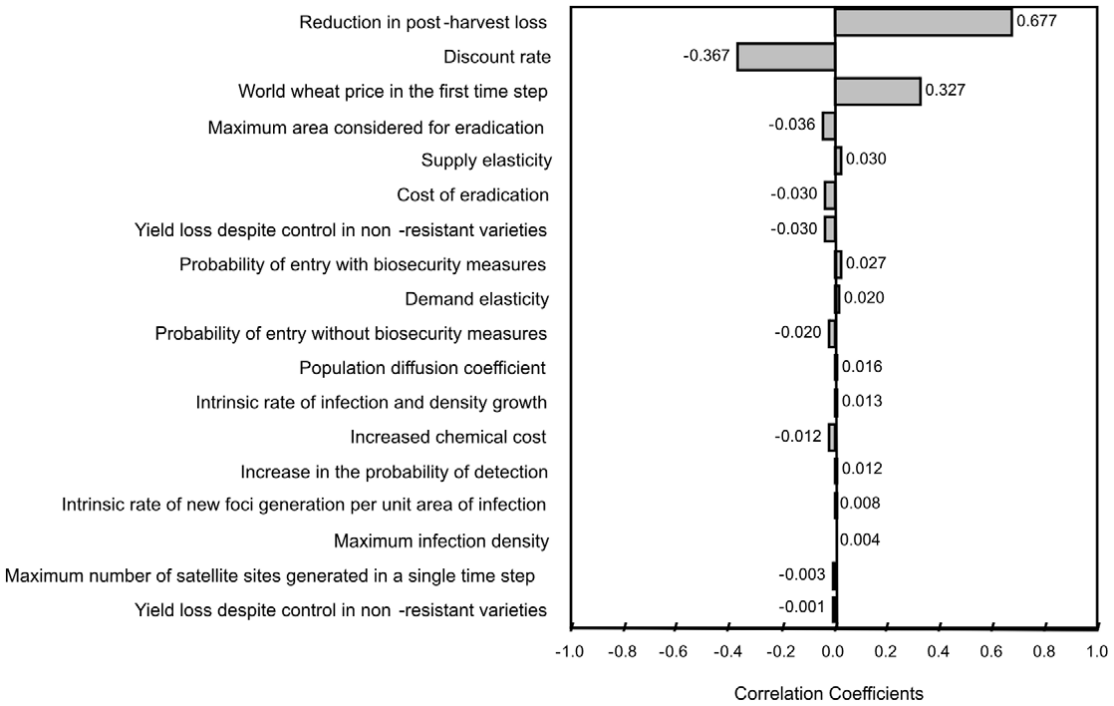
Sensitivity of results to changes in key model parameters showing Spearman rank correlation coefficients.

Given the uncertainty surrounding many of the parameters used to describe the invasion process, the sensitivity of the change in expected biosecurity benefits related to Ug99 to the key assumptions of the model was tested. Parameters were sampled from a uniform distribution with a maximum (minimum) of +50 per cent (−50 per cent) of the original values entered in to the model using Monte Carlo simulation. The Spearman's rank correlation coefficients relating the sampled model parameter values and the change in 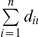 were then calculated. The results are presented in Figure 2.

The sensitivity tests indicate that the model is highly sensitive to changes in three of the 24 parameters listed in Table 2 (18 of which are shown in Figure 2). These parameters and their correlation with predicted 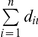 are the reduction in post-harvest loss (0.677), the discount rate (−0.367) and the world wheat price in the first time step (0.327). While post harvest losses can be strongly influenced by the biosecurity policies of wheat producing countries, the other sensitive parameters cannot be manipulated by policy makers.

## Discussion

In terms of pre-border biosecurity, our analysis predicts the spread of just one of thousands of IAS capable of reducing wheat supplies worldwide. Moreover, wheat is only one commodity of many relied on for sustenance by human populations, albeit an important one. Future research is needed to supplement our results with similar impact assessments for diseases such as the aforementioned Black Sigatoka (*M. fijiensis*) and Rice Blast (*M. oryzae*) examples. Consideration of how pest and disease impacts might change under climate change scenarios is also needed in which particular attention is paid to the possible effects of intensification and climate tolerance in crop varieties on future pest and disease fitness.

Despite the relative simplicity of the analysis, our results are indicative of the potentially huge benefits of investing in pre-border biosecurity. Ug99 is currently absent from the 15 countries used in our model, and we have used conservative estimates of the probabilities of arrival and establishment in each over time. However, in the absence of any post-border biosecurity, the estimated benefits of pre-border biosecurity measures that lower the probability of Ug99 spreading throughout the world's major wheat growing regions exceeds $US0.7 billion per annum.

Although often overlooked in the context of food security, our results clearly demonstrate the significant effect post-harvest losses exert on the global food supply. While there are few dramatic steps that can be taken to eliminate these losses, the benefits of even subtle changes could be extremely large when amplified globally. If wheat losses in storage and in transit were reduced by 5 per cent, we estimate this would generate global benefits in excess of US$135 billion over a 30 year period.

But the notion of protecting produce after it has been harvested and before it reaches a market has often been ignored as means of reducing food insecurity despite huge food losses. It has been estimated that almost 30 per cent of spoilage occurs post-harvest [6], [8]. In some countries, post-harvest crop losses to vertebrate IAS can exceed 50 per cent when conditions are favourable [10]. Lucia and Assennato [43] and Neethirajan et al. [44] estimate that 10–15 per cent loss of stored grains in India is common. Across the entire Asian continent, rats alone consume approximately 6 per cent of the annual rice harvest [10].

Given the extent of these losses and the sensitivity of our results to post-harvest loss, we would expect a high return on investment in technologies that reduce post-harvest losses. However, public investment in research and development activities has been in decline over the past 30 years. In Australia, for example, a country highly dependent on its agricultural sector for food sufficiency (although decreasingly so), total public expenditure on agricultural R&D has grown from A$115 million in 1953 to almost A$730 million in 2003, but virtually all of this growth occurred prior to 1970. As a percentage of total R&D expenditure, agricultural R&D expenditure has decreased from 20 per cent in 1982 to 8 per cent in 2003. If this trend continues, the implications may be severe in terms of technological innovation in agricultural storage and transport networks.

It is also interesting to speculate as to the probable increase in movement of IAS around the world as food trade networks become increasingly interconnected. If the rate of IAS incursions is a positive function of trade volume, the rate of incursions is set to increase with the growth of global trade markets [45]. This is a particular concern given the emphasis third generation food security policies place on diversity in supply networks (see Barrett [21]). The damage caused by these introductions threatens to detract from improvements in future food security unless IAS prevention and interception methods can be dramatically improved. Without stimuli to promote biosecurity investments across food supply chains, it is difficult to see this happening any time soon.
